# Estimates of sensitivity and specificity can be biased when reporting the results of the second test in a screening trial conducted in series

**DOI:** 10.1186/1471-2288-10-3

**Published:** 2010-01-11

**Authors:** Brandy M Ringham, Todd A Alonzo, Gary K Grunwald, Deborah H Glueck

**Affiliations:** 1 Department of Biostatistics, Colorado School of Public Health, University of Colorado, Denver, Aurora, CO, USA; 2 Department of Preventive Medicine, University of Southern California, Los Angeles, CA, USA

## Abstract

**Background:**

Cancer screening reduces cancer mortality when early detection allows successful treatment of otherwise fatal disease. There are a variety of trial designs used to find the best screening test. In a series screening trial design, the decision to conduct the second test is based on the results of the first test. Thus, the estimates of diagnostic accuracy for the second test are conditional, and may differ from unconditional estimates. The problem is further complicated when some cases are misclassified as non-cases due to incomplete disease status ascertainment.

**Methods:**

For a series design, we assume that the second screening test is conducted only if the first test had negative results. We derive formulae for the conditional sensitivity and specificity of the second test in the presence of differential verification bias. For comparison, we also derive formulae for the sensitivity and specificity for a single test design, both with and without differential verification bias.

**Results:**

Both the series design and differential verification bias have strong effects on estimates of sensitivity and specificity. In both the single test and series designs, differential verification bias inflates estimates of sensitivity and specificity. In general, for the series design, the inflation is smaller than that observed for a single test design.

The degree of bias depends on disease prevalence, the proportion of misclassified cases, and on the correlation between the test results for cases. As disease prevalence increases, the observed conditional sensitivity is unaffected. However, there is an increasing upward bias in observed conditional specificity. As the proportion of correctly classified cases increases, the upward bias in observed conditional sensitivity and specificity decreases. As the agreement between the two screening tests becomes stronger, the upward bias in observed conditional sensitivity decreases, while the specificity bias increases.

**Conclusions:**

In a series design, estimates of sensitivity and specificity for the second test are conditional estimates. These estimates must always be described in context of the design of the trial, and the study population, to prevent misleading comparisons. In addition, these estimates may be biased by incomplete disease status ascertainment.

## Background

Breast cancer is the second most deadly cancer and the sixth most common cause of death among American women of all ages [1]. Widespread introduction of screening mammography has reduced breast cancer mortality [2]. Yet mammography still misses more than a quarter of all cancers and results in a 50% cumulative false positive rate after ten mammograms [3,4].

The problems with screening mammography have led researchers to look for new screening modalities. In a trial published in 2007, Lehman *et al*. [5] used magnetic resonance imaging (MRI) to screen the mammographically normal, contralateral breast of 969 women with confirmed breast cancer. They detected additional cancers in 3.1% of the women. This is a series design, in which MRI is used after a negative mammographic exam. In this population, they showed that MRI has a sensitivity of 91% and a specificity of 88%.

Series designs, such as the one Lehman *et al*. used, are common in cancer screening [5,6]. In a series design, all study participants undergo an initial screening test. Study participants receive a second test if the first test is negative, a *test if negative *design, or positive, a *test if positive *design. These designs are also referred to as *believe the positive *and *believe the negative*, respectively [7]. In this paper, we focus on the *test if negative *design used in the trial conducted by Lehman *et al*. [5].

Because the decision to conduct the second test depends on the results of the first test, estimates of sensitivity and specificity for the *second test *are conditional on the first test results. Conditional estimates may differ from unconditional estimates, which are those observed when the second test is conducted alone. Conditional estimates should not be compared to unconditional estimates since estimates from a series trial are correct only within the context of that trial. When such conditional estimates are taken out of context, researchers may make the wrong inference about screening tests.

Estimating sensitivity and specificity may be further complicated because some cases of cancer are clinically occult, and are never identified during the trial period. This problem is extremely common in cancer screening, and may occur to a large extent. For example, in Pisano *et al*. [8], 81 out of the 335 cancers were missed by both screening tests, never observed during the one year usual follow-up term, and only observed because the investigators had planned an additional follow-up period. Because the number of observed cases of cancer is the denominator of sensitivity, failure to observe this many cases would cause a strong inflation in the estimate of observed sensitivity. This is referred to as differential verification bias [9].

Screening trials are used to assess the diagnostic accuracy of screening modalities. In cancer screening, trials are often subject to differential verification bias. These trials may have a large impact on clinical decisions as to how to screen people for cancer. In the *test if negative *series design, it is important to understand the effect of 1) differential verification bias, and 2) the conditionality of Test 2 on Test 1. We provide formulae to quantify these effects for a *test if negative *screening trial design based closely on the design used in the study by Lehman *et al*. [5].

This paper is organized into the following sections: Background, Methods, Results, and Discussion. In the Methods section, we describe the single test and series screening trial designs, present our model assumptions, and define notation. In the Results section, we outline the derivation of the formulae for the observed bias in both trial designs. Also in the Results section, we explore the effect of three important factors on the observed estimates of diagnostic accuracy. In the Discussion section, we present the results in the context of previous literature and propose future avenues of research.

## Methods

We compare two screening trial designs in this paper: a single test design and a two test series design where the investigator is interested in the diagnostic accuracy of the second test. The series design we consider is a *test if negative *design, based closely on the trial of Lehman *et al*. [5].

We consider the screening studies from two points of view. The first is an omniscient point of view in which the *true *disease status is known for each participant. We also consider the point of view of the study investigator, who can only see the *observed *results of the study. The study investigator does not observe every case of disease. Cases fail to be observed if 1) both of the screening tests miss the case, and 2) the case is never diagnosed during the follow-up period. Unless a participant is diagnosed with disease during the study, the study investigator assumes that the participant is disease free. In this way, the *true *disease status can differ from the *observed *disease status.

The study investigator calculates *observed *sensitivity using the number of *observed *cases of disease in the trial as the denominator. *Observed *specificity is calculated similarly. The *observed *sensitivity and specificity estimates may not be the same as the *true *sensitivity and specificity. We quantify the bias by comparing the *true *and *observed *estimates of sensitivity and specificity. Here, we use the word "bias" in the epidemiological sense, as the difference between the observed estimates and the truth.

### Single test design

**Figure 1:**
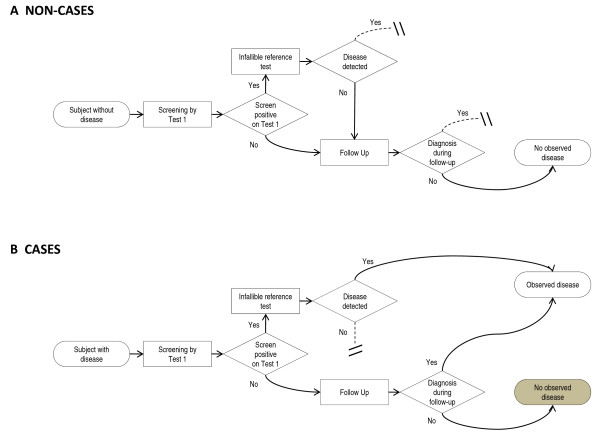
**Flowchart for single test design**. Flowchart depicts a single test screening trial from an omniscient point of view. Dashed lines indicate a pathway that is unavailable to that class of participants (true case or true non-case) due to the assumptions of our model. The gray box indicates cases that are misclassified as noncases by the study investigator.

In a single test design, all participants are screened with one test. A flowchart for this design is shown in Figure 1. The flowchart is presented from an omniscient point of view, rather than from the point of view of the study investigator. The goal is to point out where the *observed *disease status differs from the *true *disease status. If the screening test is positive, the participant undergoes a reference test, which is used to make the diagnosis. In cancer screening the two reference tests are typically follow-up, or a further diagnostic process, which may lead to biopsy. Definitive diagnosis of cancer is made only through biopsy and pathologic review, which we assume to be 100% sensitive and specific.

In general, two sorts of mistakes can occur in screening trials. The study investigator can declare that participants have disease when they do not, or the study investigator can miss cases of disease. In this trial, as shown in Figure 1, only the second sort of mistake occurs. Missed cases occur because only some participants receive biopsy, and definitive disease status ascertainment. Because the biopsy is invasive and can be done only if a lesion is observed, it is unethical and infeasible to do a biopsy unless there are suspicious screening test results. Instead, participants who have normal screening test results enter a follow-up period. At the completion of the follow-up period, participants who have normal results on all screening tests are assumed to be disease-free. This assumption may be wrong. Some participants who are assumed to be disease free may actually have disease. Because the method of disease ascertainment differs depending on the outcome of the index test, the trial is subject to differential verification bias [9]. Differential verification bias leads to overestimates of both the sensitivity and specificity [10].

During follow-up, some participants may choose to have a procedure that will allow diagnosis, even without a suspicious test result. For example, in breast cancer screening studies, women at perceived risk may choose to undergo prophylactic mastectomy. Elective procedures, like prophylactic mastectomy, do not occur as a planned part of a screening study. However, elective procedures do allow additional, and possibly random, ascertainment of disease status.

### Test if negative series design

**Figure 2:**
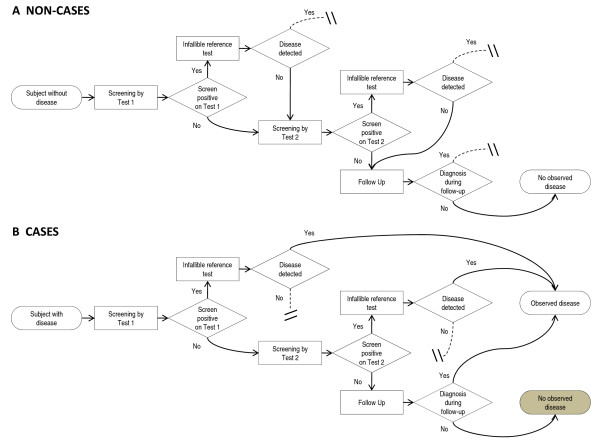
**Flowchart for test if negative series design**. Flowchart depicts a *test if negative *series screening trial from an omniscient point of view. Dashed lines indicate a pathway that is unavailable to that class of participants (true case or true non-case) due to the assumptions of our model. In A, non-cases who screen positive on Test 1 are given a reference test. The results of this test are negative. The study investigator then goes on to screen the participant with Test 2, in case the reference test has failed. In B, cases who screen positive on Test 1 are given a reference test. The results of this reference test are positive and the study participant is observed to have disease. The gray box indicates cases that are misclassified as non-cases by the study investigator. The design is similar to that of Lehman *et al*. [5].

The flowchart for the *test if negative *series design is shown in Figure 2. The flowchart is presented from an omniscient point of view. The *test if negative *design described below is modeled after the trial conducted by Lehman *et al*. [5]. In the *test if negative *design each participant is screened with a first screening test (Test 1). Participants who have negative results on the first screening test are given a second screening test (Test 2). Participants also get a second screening test if the first screening test is positive, but the biopsy is negative. If either the first or second test result is positive, a reference test is used to ascertain the disease status. Participants who are negative on both screening tests are followed for a defined time period. Since we model this design on the trial conducted by Lehman *et al*. [5], we do not expect women will develop signs and symptoms during this period. All women in the trial conducted by Lehman *et al*. [5] were undergoing systemic therapy for cancer in their first breast, which suppresses any occult cancer in the contralateral breast. However, there is a chance participants will choose to mitigate their risk through prophylactic mastectomy, a procedure which allows determination of their disease status and leads to diagnosis during the follow-up period. Participants who have two negative screening tests, and who are not diagnosed during the follow-up period are assumed to be disease-free. Like the single test design, the *test if negative *design can result in missed cases of disease but no false diagnoses.

### Assumptions, notation, and definitions

We assume that the goal of the investigator is to estimate the diagnostic accuracy of Test 2. It is important portant to point out that this was not the stated goal of the trial published by Lehman *et al*. [5]. However, estimating the diagnostic accuracy of MRI is one possible use of their results.

We make four additional, simplifying assumptions for our model: 1) the screening test results for different study participants are independent, 2) the chance that a participant screens positive on each screening test depends only on disease status, 3) the reference test given to participants who screen positive is 100% sensitive and specific, and 4) participants will not spontaneously show signs and symptoms during follow-up but may elect to have a procedure that allows ascertainment of their true disease status. The elective procedure occurs randomly, rarely, and independently of the screening test results.

Results of the first and second screening tests, Test 1 and Test 2, are *T*_1 _and *T*_2_, respectively. The proportion of true cases in the sample is denoted by *p_D_*. The proportion of participants in the sample who undergo an elective procedure or have a similarly definitive evaluation of the breast is denoted *p*_*E*_. We define the proportion of cases that test negative on Test 1 as *FN*(1), the proportion of cases that test positive on Test 1 as *TP*(1), the proportion of non-cases that test negative on Test 1 as *TN*(1), and the proportion of non-cases that test positive on Test 1 as *FN*(1) Similar notation is used for Test 2. *FN*(1, 2) is the proportion of cases that test negative on both Test 1 and Test 2, or the double negative cases. *FN*(1, 2) is a measure of agreement between Tests 1 and 2. Sensitivity is defined as the proportion of cases that screen positive out of all cases [[11]; pg. 15]. Specificity is defined as the proportion of non-cases that screen negative out of all non-cases [[11]; pg. 15]. *TP*(1) and *TN*(1) are the true sensitivity and specificity of Test 1, respectively. *TP*(2) and *TN*(2) are the true sensitivity and specificity of Test 2, respectively.

## Results

We present formulae for the observed sensitivity and specificity for Test 2 in the single test and *test if negative *trial designs.

### Single test design

All possible outcomes of the single test design are shown in Table 1. We refer to the single test as Test 2, since we are going to compare it to the second test in a series design. Table 2 provides the probability of each cross-classification of test result and true disease status that can occur.

**Table 1:** Outcomes for a single-test cancer screening trial

True Disease Status	Test 2 Result	Elective Procedure	Observed Disease Status
+	+	--	+
+	-	Yes	+
+	-	No	-^†^
-	+	Yes	-
-	+	No	-
-	-	Yes	-
-	-	No	-

A double dash indicates an event that will not occur under the assumptions of our model. ^†^Cases misclassified as non-cases.

**Table 2:** True disease status and Test 2 results in a single test trial design

		True Disease Status	
		+	-
Test 2	+	*p*_*D*_*TP*(2)	(1 - *p*_*D*_)*FP*(2)
	-	*p*_*D*_*FN*(2)	(1 - *p*_*D*_)*TN*(2)

Table 3 gives the probability of each combination of test result and observed disease status that can occur.

**Table 3:** Observed disease status and Test 2 results in a single test trial design

		Observed Disease Status	
		+	-
Test 2	+	*p*_*D*_*TP*(2)	(1 - *pD*)*FP*(2)
	-	*p*_*D*_*p*_*E*_*FN*(2)	*p*_*D*_(1 - *p*_*E*_)*FN*(2)+(1 - *p*_*D*_)*TN*(2)

The observed sensitivity, sens(*O*), for a single test design is(1)

with bias specificity, spec(*O*), given by(2)

The bias in observed sensitivity for the single test design, *B*_*sen*(*O*)_, is the difference between the observed and true sensitivity. The percent bias in sensitivity is 100 [*B*_*sens*(*O*)_/*TP*(2)].

Calculations are similar for specificity.

### Test if negative series design

All possible outcomes of a *test if negative *series design are shown in Table 4.

**Table 4:** Outcomes for a test if negative cancer screening trial

True Disease Status	Test 1 Result	Test 2 Result	Elective Procedure	Observed Disease Status
+	+	--	--	+
+	-	+	--	+
+	-	-	Yes	+
+	-	-	No	-^†^
-	+	+	Yes	-
-	+	+	No	-
-	+	-	Yes	-
-	+	-	No	-
-	-	+	Yes	-
-	-	+	No	-
-	-	-	Yes	-
-	-	-	No	-

A double dash indicates an event that will not occur under the assumptions of our model. ^†^Cases misclassified as non-cases.

Table 5 is the probability of each combination of test result and observed disease status that can occur for the *test if negative *series design. These results are dependent on the quantity, *Q*, the probability that a participant proceeds to Test 2 after being screened with Test 1. The quantity, *Q *is the sum of two probabilities: 1) the probability that a participant screens negative on Test 1, and 2) the probability that a non-case screens positive on Test 1. The sum simplifies to(3)

**Table 5:** Observed disease status and Test 2 results in a test if negative trial design

Test 2	Observed Disease Status	Probability
+	+	*p*_*D *_[*FN*(1) - *FN*(1, 2)]/*Q*
+	-	(1 - *p*_*D*_)*FP*(2)/*Q*
-	+	*p*_*D*_*p*_*E*_*FN*(1,2)/*Q*
-	-	[(1 - *p*_*D*_)*TN*(2) + *p*_*D*_(1 - *p*_*E*_)*FN*(1, 2)/*Q*

The observed sensitivity for the series design, sens(*O*^-^), is(4)

Note that the observed sensitivity, like the true sensitivity, does not depend on the disease prevalence since *p*_*D *_cancels from both the numerator and denominator. Observed specificity, spec(*O*^-^), is given by(5)

The bias in the observed sensitivity for the series design,  is the difference between the observed and true sensitivity. The percent bias in sensitivity is 100 [/TP(2)]. Calculations are similar for specificity.

### Three factors affecting bias

Our results demonstrate that the amount of bias is affected by three factors: 1) disease prevalence, 2) the proportion of study participants who undergo an elective procedure, and 3) the chance that the two tests miss the same case. The bias arises from two sources, the series design and the lack of complete disease status ascertainment.

**Figure 3:**
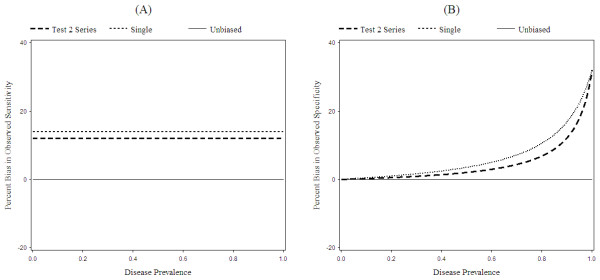
**Effect of disease prevalence on percent bias**. Effect of disease prevalence on percent bias in observed sensitivity (A) and specificity (B). Parameter definitions are as in "Parameters" (Table 6), except that the disease prevalence is allowed to vary. Percent bias is the bias in observed sensitivity or specificity divided by the true sensitivity or specificity. The observed results for Test 2 in a *test if negative *series design are denoted by "Test 2 Series". The observed results for a single test design are denoted by "Single". The observed sensitivity is biased upwards by 14% for the single test design and 12% for the series design.

**Figure 4:**
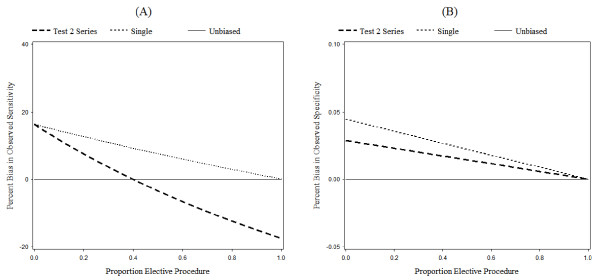
**Effect of proportion elective procedure on percent bias**. Effect of the proportion of participants who undergo an elective procedure on percent bias in observed sensitivity (A) and specificity (B). Note that the scale of the y-axis of the specificity graph (B) is enlarged to show minute changes. Parameter definitions are as in "Parameters" (Table 6), except that the proportion elective procedure is allowed to vary. Otherwise as Figure 3.

**Figure 5:**
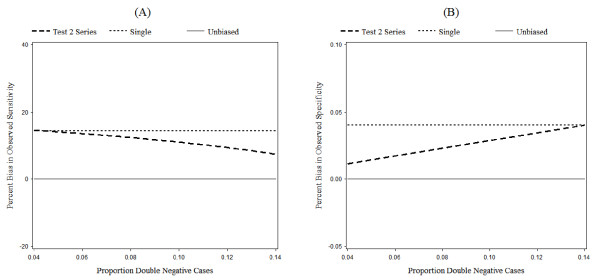
**Effect of proportion of double negative cases on percent bias**. Effect of the proportion of double negative cases on percent bias in observed sensitivity (A) and specificity (B). Note that the scale of the y-axis of the specificity graph (B) is enlarged to show minute changes. Parameter definitions are as in "Parameters" (Table 6), except that the proportion double negative cases is allowed to vary. Otherwise as Figure 3.

Figures 3, 4, and 5 show the percent bias in the observed sensitivity and specificity under different assumptions. In these graphs, we show three lines. The first line, "Unbiased", represents the true sensitivity and specificity of Test 2. The second line, "Single", represents the observed results for a screening trial with only one screening test. The third line, "Test 2 Series", represents the observed results for Test 2, when Test 2 is the second of two tests conducted in series.

Parameter definitions for each of the plots are as in "Parameters" (Table 6), except that the indicated parameter of interest is allowed to vary. The parameters were chosen to represent a realistic cancer screening trial with low disease prevalence and low disease status ascertainment during follow-up. We chose the true sensitivity and specificity of Test 1 and Test 2 to approximate the diagnostic properties of mammography and MRI, respectively [12,13]. We chose the proportion of double negatives based on those seen in Lehman *et al*. [5]. In the trial conducted by Lehman *et al*. [5], 3 out of 33 women had cancers that were missed by both mammography and MRI screening.

**Table 6:** Parameters

Parameter	Chosen Value
*p*_*D*_	0.01
*p*_*E*_	0.10
*TP*(1)	0.69
*FN*(1)	0.31
*FP*(1)	0.24
*TN*(1)	0.76
*TP*(2)	0.86
*FN*(2)	0.14
*FP*(2)	0.24
*TN*(2)	0.76
*FN*(1, 2)	0.09

Each graph shows that the observed sensitivity and specificity of Test 2 for both the single test and series designs are inflated relative to the true sensitivity and specificity, though there is less inflation for the series design. Estimates for both the single test and series designs are biased upward due to differential verification bias [10]. Differential verification bias arises when some true cases are misclassified as non-cases because they never receive definitive disease status ascertainment [9]. We refer to the missed cases as "misclassified cases". Estimates for the series design are lower than those for the single test design because, in the series design, only a portion of the cases, the Test 1 false negatives, proceed to Test 2. We refer to the portion of cases that do not proceed to Test 2 as the "absent cases". The numerator and denominator of the sensitivity of Test 2 in the series design are decreased by the same number, that is, the number of absent cases. The numerator decreases proportionately more than the denominator since it is smaller, which results in an overall decrease in sensitivity. The same phenomenon occurs for the observed specificity since it includes misclassified cases, some of which become absent cases in the series design.

### Disease prevalence

Figure 3 shows the relationship between the percent bias in the observed sensitivity and specificity and disease prevalence. The bias in the observed sensitivity is unaffected by disease prevalence. The bias in observed specificity, however, increases with increasing disease prevalence.

Observed specificity increases with disease prevalence because both the numerator and denominator of the observed estimates of specificity include misclassified cases. As the disease prevalence increases, so does the number of misclassified cases. A larger number of misclassified cases increases both the numerator and denominator of observed specificity, though the numerator increases proportionately less since it is numerically smaller than the denominator. The overall effect is an increase in the observed specificity.

### Proportion elective procedure

Figure 4 shows the relationship between the percent bias in the observed sensitivity and specificity and the proportion of participants who undergo an elective procedure. As more participants undergo an elective procedure, the bias in the observed sensitivity and specificity for both the single test and series designs decreases.

As the proportion of participants who undergo an elective procedure increases, the number of misclassified cases decreases. These cases are detected by the elective procedure, not by the test. This causes the denominator of observed sensitivity to increase while the numerator remains constant.

In Figure 4A, as the proportion elective procedure increases to one, the observed sensitivity for the single test design decreases to the true sensitivity. The observed sensitivity of Test 2 for the series design, however, falls below the true sensitivity. As the proportion of participants who undergo an elective procedure increases, the deflation in observed sensitivity caused by the absent cases eventually outweighs the inflation caused by the missing cases. As a result, the observed sensitivity of Test 2 in the series design drops below the true sensitivity and the percent bias goes from positive to negative.

The relationship between the proportion of participants who undergo an elective procedure and the observed sensitivity of Test 2 in the series design leads to an important observation. When a large number of cases are diagnosed during the follow-up period, the effect of the conditionality of Test 2 on Test 1 will have a greater influence on the estimates of observed sensitivity for Test 2 than differential verification bias.

This plot (Figure 4B) also shows that the observed specificity very slightly decreases as the proportion of participants who undergo an elective procedure increases. When few study participants undergo an elective procedure, there are more misclassified cases. Thus, the observed specificity is inflated compared to the true specificity.

Figure 4B shows the effect of proportion elective procedure on the observed specificity using an enlarged scale for the y-axis. The magnitude of the effect of proportion elective procedure on the observed specificity is very small due to the low disease prevalence.

### Proportion double negative

Figure 5 shows the relationship between the percent bias in observed sensitivity and specificity and the proportion of cases that screen negative on both tests, or the proportion of double negative cases. In general, as the proportion of double negatives increases, the percent bias in observed sensitivity for the series design decreases and the percent bias in observed specificity increases.

Recall that differential verification bias inflates sensitivity. The series design slightly reduces that bias. As the proportion of double negative cases increases, the proportion of true positives on Test 2 decreases since more and more cases screen negative. This causes the observed sensitivity of Test 2 in the series design to decrease, while the observed sensitivity in the single test design remains constant (Figure 5A).

In Figure 5B, the percent bias in the observed specificity of Test 2 in the series design very slightly increases as the proportion of double negative cases increases. Recall that, in the *test if negative *series design, all non-cases will proceed to Test 2. The observed specificity depends on the proportion of misclassified cases. As the proportion of double negative cases increases, more of the cases who tested negative on Test 1 will also test negative on Test 2. As a result, there will be more misclassified cases and the observed specificity of Test 2 for the series design will increase. Note that the change in observed specificity in Figure 5B is very small. As in Figure 4B, this is because the disease prevalence is very small, which results in a large number of noncases relative to cases.

## Discussion

In this paper, we discuss the bias that can arise in cancer screening trials due to incomplete disease status ascertainment in a *test if negative *series trial design. The design we considered was modeled closely after a recently completed and published trial by Lehman *et al*. [5]. The goal of this trial was to assess the diagnostic yield of MRI over mammography. It was not to assess the diagnostic accuracy of MRI for comparison to other screening modalities. However, it is easy to take the results of the trial out of context. Other researchers may be tempted to cite their results as historic estimates of the diagnostic accuracy of MRI or emulate the *test if negative *trial design to estimate the diagnostic accuracy of Test 2. It is, therefore, important to explore the effects of the *test if negative *trial design on the estimates of the diagnostic accuracy of Test 2.

Although we modeled our design on real trials, we made simplifying assumptions. We assumed that biopsy was essentially infallible. In real cancer studies, even biopsy makes diagnostic errors. In addition, we assumed that no study participant would show signs and symptoms of disease, because they were receiving systemic therapy. In fact, recurrences of cancer and new primary cancers can occur even during chemotherapy and radiation.

We have been unable to find other research that simultaneously considers how conditioning and incomplete disease status ascertainment affect estimates of sensitivity and specificity. The majority of literature focuses on estimating the accuracy of a diagnostic program comprising several tests [7,14-16]]. In contrast, we are interested in estimating the diagnostic accuracy of the in *second test *a series of two tests. Most authors also assume that the true disease status of each participant is known [7,14-16]]. We do not make this assumption, as it is unlikely to be true in cancer screening trials.

Rutjes *et al*. [9] provides a thorough discussion of the pitfalls faced by clinicians when evaluating medical tests in the absence of a true gold standard. Whiting *et al*. [10] also catalogues biases that can occur in screening trials. Neither Rutjes *et al*. [9] nor Whiting *et al*. [10] discuss the additional effect of using a series screening trial design to estimate diagnostic accuracy.

Lehman *et al*. [5] point out that the estimated diagnostic accuracy of MRI is higher in their study than in other published studies. They posit that this could be due to advances in breast cancer screening technology and increased skill at analyzing imaging results. As noted in this paper and in the papers by Whiting *et al*. [10] and Rutjes *et al*. [9], biases resulting from trial design may also cause an inflation in the observed estimates of diagnostic accuracy. While the results of the trial conducted by Lehman *et al*. [5] may have been affected by differential verification bias, we suspect that the results were not affected by bias due to the conditionality of Test 2 (MRI) on the results of Test 1 (mammography). We give our rationale below.

The figures presented in the results section use parameters that are consistent with what we would expect for the trial conducted by Lehman *et al*. [5]. Using the parameter values estimated from this trial and the formulae presented in this paper, we calculated the percent bias in the observed sensitivity and specificity for each trial design. The percent bias in the observed specificity of Test 2 relative to the true specificity is near zero. However, the percent bias in the observed sensitivity of Test 2 relative to the true sensitivity is 14% for the single test design and 12% for the series design. Since there is little difference between the single test and series designs, the detected upward bias is mainly due to differential verification of disease status, rather than the conditionality of MRI on the results of mammography.

In some circumstances, the *test if negative *trial design may be the best choice available, due to external constraints. An investigator can use the formulae presented in this paper to conduct a sensitivity analysis of their estimates of the diagnostic accuracy of Test 2. For the trial conducted by Lehman *et al*. [5], an example of this sort of sensitivity analysis is given in the immediately preceding paragraph. The investigator can choose a range of reasonable values for the disease prevalence, the proportion of participants who undergo an elective procedure, and the agreement between Test 1 and 2 results for cases, in order to place bounds on the amount of bias that may arise from their choice of study design. An investigator may be able to directly estimate the portion of bias due to differential verification by estimating the number of missing cases. This number can be estimated by looking at the number of participants who are determined to be cases out of those who tested negative on both tests and chose to undergo an elective procedure. In practice, as the percentage of subjects who choose an elective procedure is usually low, the stability of this estimate may be questionable.

Aside from the series trial design, there are two further characteristics of the trial conducted by Lehman an *et al*. [5] that should be noted. First, the results of the trial are presented per breast, rather than per lesion, which is more common [8,12,17]. Second, all of the participants in the trial had already developed cancer in one breast before being screened for cancer in the second breast. The development and treatment of cancer in that first breast will affect screening practices and treatment of the second breast. For example, when screening the contralateral breast, we noted that participants are less likely to show signs and symptoms during follow-up since they are undergoing systemic therapy for cancer in the first breast.

In this paper, we have shown that estimates of diagnostic accuracy for the second test in *test if negative *series screening trials with incomplete disease status ascertainment can be subject to bias. Glueck *et al*. [18], showed a similar bias in screening studies conducted in parallel. If both designs are flawed, what design should be adopted by researchers seeking to characterize screening modalities? The answer is unclear. Because screening trials affect the health of millions of people, methods for bias correction for both parallel and series screening trial designs are needed.

## Conclusions

We have shown that estimates of diagnostic accuracy for the second test in a *test if negative *screening trial are different than estimates obtained from a trial design that utilizes only a single test. Because of this, researchers must be careful to always cite estimates of diagnostic accuracy within the context of the trial that supplied them. Observed estimates of the diagnostic accuracy are also subject to differential verification bias because some cases do not receive definitive disease status ascertainment. Further research is needed to derive methods to 1) obtain unconditional results from a series trial design, and 2) correct for differential verification bias.

## Competing interests

The authors declare that they have no competing interests.

## Authors' contributions

BMR conducted the literature review, developed the mathematical framework, derived the results, wrote computer programs, produced graphs, and prepared the manuscript. TAA provided advice on how to relate the topic to previous work in the field. TAA and GKG reviewed the work and gave important editorial suggestions that greatly improved the manuscript. DHG suggested the topic, and guided the development of the work. All authors have read and approved the final manuscript.

## Pre-publication history

The pre-publication history for this paper can be accessed here:

http://www.biomedcentral.com/1471-2288/10/3/prepub
